# PEC/Colorimetric Dual-Mode Lab-on-Paper Device via BiVO_4_/FeOOH Nanocomposite In Situ Modification on Paper Fibers for Sensitive CEA Detection

**DOI:** 10.3390/bios13010103

**Published:** 2023-01-06

**Authors:** Xu Li, Jiali Huang, Jiayu Ding, Mingzhen Xiu, Kang Huang, Kang Cui, Jing Zhang, Shiji Hao, Yan Zhang, Jinghua Yu, Yizhong Huang

**Affiliations:** 1 School of Chemistry and Chemical Engineering, University of Jinan, Jinan 250022, China; 2 School of Materials Science and Engineering, Nanyang Technological University, Singapore 639798, Singapore; 3 School of Materials Science & Engineering, Dongguan University of Technology, Dongguan 523808, China; 4 Key Laboratory of Optic-Electric Sensing and Analytical Chemistry for Life Science, MOE, Qingdao University of Science and Technology, Qingdao 266042, China

**Keywords:** PEC, colorimetric, lab-on-paper device, BiVO_4_/FeOOH, CEA

## Abstract

A dual-mode lab-on-paper device based on BiVO_4_/FeOOH nanocomposites as an efficient generating photoelectrochemical (PEC)/colorimetric signal reporter has been successfully constructed by integration of the lab-on-paper sensing platform and PEC/colorimetric detection technologies for sensitive detection of carcinoembryonic antigen (CEA). Concretely, the BiVO_4_/FeOOH nanocomposites were in situ synthesized onto the paper-working electrode (PWE) through hydrothermal synthesis of the BiVO_4_ layer on cellulose fibers (paper-based BiVO_4_) which were initially modified by Au nanoparticles for improving the conductivity of three dimensional PWE, and then the photo-electrodeposition of FeOOH onto the paper-based BiVO_4_ to construct the paper-based BiVO_4_/FeOOH for the portable dual-mode lab-on-paper device. The obtained nanocomposites with an FeOOH needle-like structure deposited on the BiVO_4_ layer exhibits enhanced PEC response activity due to its effective separation of the electron–hole pair which could further accelerate the PEC conversion efficiency during the sensing process. With the introduction of CEA targets onto the surface of nanocomposite-modified PWE assisted by the interaction with the CEA antibody from a specific recognition property, a signal-off PEC signal state with a remarkable photocurrent response decreasing trend can be achieved, realizing the quantitative detection of CEA with the PEC signal readout mode. By means of a smart origami paper folding, the colorimetric signal readout is achieved by catalyzing 3,3′,5,5′-tetramethylbenzidine (TMB) to generate blue oxidized TMB in the presence of H_2_O_2_ due to the satisfied enzyme-like catalytic activity of the needle-like structure, FeOOH, thereby achieving the dual-mode signal readout system for the proposed lab-on-paper device. Under the optimal conditions, the PEC and colorimetric signals measurement were effectively carried out, and the corresponding linear ranges were 0.001–200 ng·mL^−1^ and 0.5–100 ng·mL^−1^ separately, with the limit of detection of 0.0008 and 0.013 ng·mL^−1^ for each dual-mode. The prepared lab-on-paper device also presented a successful application in serum samples for the detection of CEA, providing a potential pathway for the sensitive detection of target biomarkers in clinical application.

## 1. Introduction

Carcinoembryonic antigen (CEA), a broad-spectrum tumor marker, is named for its early discovery in red bowel cancer and fetal malignancy [1,2]. It is known that CEA is mainly distributed in the digestive system cancer of embryonic leaf origin and the digestive duct tissue of normal embryos in human body. Generally, a tiny amount of CEA distributed in the serum of normal people could reflect the position of various tumors in the human body [3]. Accordingly, sensitive detection of human blood CEA content is normally used for colorectal cancer, breast cancer, lung cancer efficacy judgment, disease development, monitoring, and prognosis estimation [4]. Meanwhile, accurate determination of CEA content in sera at an anterior period is practical for the well-timed medical therapy of potentially cancered people [5]. Hence, it is crucial to explore a reliable, noninvasive, and effective strategy for the ultrasensitive assay of CEA based on various novel and developed techniques. To date, many analytical techniques have been exploited for ingenious bioassay of CEA, such as electrochemiluminescence (ECL) [5], surface-enhanced Raman scattering (SERS) [6], fluorescence (FL) [7], and electrochemistry (EC) [8,9]. These analytical efforts usually require complex steps, costly equipment, and have a high time cost. Thus, to meet the requirements of real sample bioanalysis, it is urgent to explore novel strategies with efficient techniques for the ultrasensitive biosensing of CEA. Photoelectrochemical (PEC) biosensor, as a burgeoning sensing technology, pyramidally demonstrated its advantages, such as, simplified analysis operations, abbreviated and inexpensive instrumentation, and ease of miniaturization for biological detection applications in recent times [10].

In principle, PEC technology developed from electrochemistry technology, which normally involves the process of light transformation into electricity, and chemical/electric energy interconversion. Compared with the traditional electrochemical system, the PEC technology generally contains two separated provenience units towards light response excitation and electrical signal readout, providing a more superior sensitivity analysis means in respect to the lessened back noise [11,12,13,14,15,16]. To realize a sophisticated PEC detection system, electron/hole pairs separation and the electricity diversion process normally occurs on the light-sensitive substance in the case of light radiation, giving rise to electro-optic semaphores switching and, ulteriorly, eliciting an arresting differentiation under the consistent light reception, which is generally resolved by the capabilities of light-sensitive substances [17,18]. Accordingly, to carry out an efficient and sensitive PEC system regarding the assay of CEA targets, it is considerably significant to explore photoactive materials processing high carrier mobilities, valid band gap, and applicable corrosion resistance.

Semiconductor materials, as a typical photoactive material, are normally utilized in the construction of PEC biosensors, because they could generate photogenerated carriers (electron/hole) under external light to achieve photoelectric conversion. In the last few years, various semiconductor materials have served as photoactive materials to construct different kinds of PEC biosensors, for instance, ZnO [19,20], TiO_2_ [21], CdS [22], Cu_2_O [23], and WO_3_ [24]. Among the various semiconductor materials, BiVO_4_ is considered as a suitable photoanode material because of its suitable band gap (~2.4 eV), high chemical stability, low toxicity, and earth abundance [25,26,27]. However, the photoanode prepared only from BiVO_4_ has an undesirable photocurrent response due to the limited light absorption, surface charge recombination, and poor electron transfer efficiency [28,29,30,31]. Therefore, it is important to find a material that can improve the PEC response of BiVO_4_. In order to obtain a higher photocurrent, improvements can be made in the following three aspects: (1) the light absorption range of the photoactive material; (2) the separation efficiency of the photoinduced carriers; and (3) the surface modification engineering of the photoelectrode [32]. Generally, introducing extra material with unique photoactive properties to form a nanocomposite is one of the potential strategies to improve the performance of PEC biosensors [33]. The FeOOH has wide application prospects in many fields because of its merits of environmental friendliness, satisfied photocatalytic activity, low cost, natural abundance, and so on [34]. The modification of FeOOH on the surface of BiVO_4_ is beneficial to enhance the migration of photoinduced charge, exhibit stronger light response capacity, and reduce the recombination of electron–hole pairs [35,36,37,38]. Moreover, the FeOOH with excellent enzyme-like catalytic activity could catalyze 3,3′,5,5′-tetramethylbenzidine (TMB) to generate blue oxidized TMB [39,40,41]. In this way, colorimetric detection could be integrated with PEC detection to meet the end-user requirement of point-of-care analysis.

To achieve the purpose of integrating PEC and colorimetric detection, a sensing platform supported by a suitable substrate with the ability of rapid, easy operation, and direct readout design should be thoroughly considered. Since a three-dimensional (3D) microfluidic device was constructed onto a cellulose paper sensing platform in 2007 at Whitesides Research Group, lab-on-paper devices with stacking paper slip layers have attracted considerable interest because of their potential multifunctionality when applied in bioassay device exploration. For example, the cellulose paper was applied as a flexible platform to prepare portable bioassay devices, and even dual-mode lab-on-paper biosensors based on its inherent merits of paper, such as bendable, low cost, and desirable biocompatibility and hydrophilicity [42,43,44,45,46,47,48,49,50]. Until now, lab-on-paper sensor platforms combined with different analytical technologies have been applied to construct various lab-on-paper biosensors, such as the PEC lab-on-paper device [51], the electrochemical lab-on-paper device [52], the electrochemiluminescence lab-on-paper device [53], the visual lab-on-paper device [54,55], and even the dual-mode lab-on-paper device [56,57,58,59]. The above reported literature indicates that the flexible cellulose paper with desirable properties is a satisfactory substrate for integration of PEC and colorimetric techniques to achieve CEA sensitive detection. Moreover, the dual-mode detection method through the combination of two different assay technologies is able to realize accurate screening and rapid quantitation of the target biomarkers. For example, the dual-mode bioassay strategies were proved to possess a more efficient and sensitive performance than the single mode tactic [60,61]. The dual-mode biosensor, such as PEC technology combined with the colorimetric analytical method, has been developed for the diagnosis of cancer biomarkers from clinical samples on account of its advantages, such as high sensitivity, fast response, and ease of operation [57,61].

**Scheme 1:**
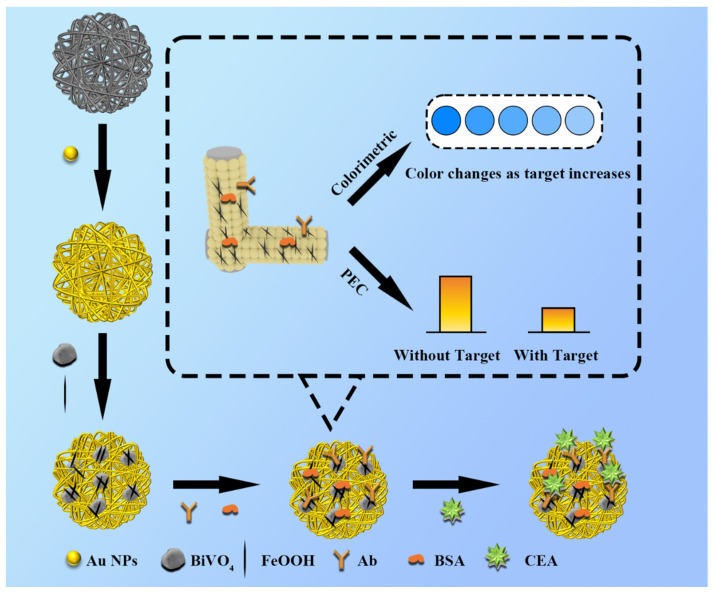
Schematic illustration of the preparation for in situ growth of BiVO_4_/FeOOH nanocomposites onto cellulose fibers to construct lab-on-paper biosensing platform and the dual-mode signal readout.

Herein, a dual-mode lab-on-paper device based on BiVO_4_/FeOOH nanocomposites was fabricated to realize ultrasensitive PEC/colorimetric detection of CEA targets. The FeOOH/BiVO_4_ nanomaterials were in situ modified onto the surface of cellulose fibers for the first time, where a uniform layer of Au nanoparticles (NPs) was grown for endowing the high electron transport rate, to obtain the higher initial photocurrent response. After the FeOOH nanoneedles were modified on the surface of the BiVO_4_ layer via the photo-electrodeposition approach, the photocurrent response could be significantly enhanced due to the prepared compound of FeOOH/BiVO_4_ improving the migration of photogenerated carriers and reducing the recombination of the photoinduced electron–hole, further resulting in significant improvement of the PEC response compared with a pure BiVO_4_ photoelectrode. After modification of CEA antibodies (Ab) on the surface of the FeOOH/BiVO_4_-modified paper working electrode (PWE) assisted with the BSA molecules which were introduced to avoid nonspecific interaction, the biomarker (CEA) can be specifically recognized and linked via the Ab onto the interface of the FeOOH/BiVO_4_ composites, leading to a decline of the PEC signal, thereby realizing the ultrasensitive PEC detection of CEA targets. Meanwhile, the nanocomposite of FeOOH/BiVO_4_ exhibits peroxidase-like activity, which could cause oxidation of TMB to produce a blue product in the presence of H_2_O_2_, realizing an obvious visual detection of CEA (Scheme 1). Consequently, a dual-mode lab-on-paper device based on enhanced PEC active FeOOH/BiVO_4_ nanocomposites placed onto cellulose fibers was successfully constructed and was able to achieve ultrasensitive detection of CEA by integrating PEC and colorimetric bioassay. We believe that our present work could not only meet the requirement of point-of-care detection, but also open a way to fabricate biosensors for biomarkers detection.

## 2. Experimental Section

### 2.1. Reagents and Apparatus

All aqueous solutions were prepared with deionized water (DI water, resistivity ≥18.25 MΩ cm), which was obtained from a Lichun water-purification system. Bi(NO_3_)_3_·5H_2_O was provided by Aladdin (Shanghai, China). Fe_3_O_4_, KCl, NH_4_VO_3_, and ascorbic acid (AA) were obtained from Maclean Biochemical Technology Co., Ltd. (Shanghai, China). Acetic acid, urea, and 3,3′,5,5′-Tetramethylbenzidine (TMB) were purchased from Shanghai Jizhi Biochemical Technology Co., Ltd. (Shanghai, China). Chloroauric acid (HAuCl_4_·4H_2_O) was ordered from Sangon Biological Engineering Technology and Services Co., Ltd. (Shanghai, China). Bovine serum albumin (BSA) was obtained from Sigma-Aldrich Chemical Co. Phosphate-buffered saline (PBS, pH 7.4, 0.1 M) was prepared with Na_2_HPO_4_, KH_2_PO_4_, and KCl. Glutaraldehyde (GLD; 50%) was purchased from Tianjin Chemical Plant Co., Ltd. (Tianjin, China). Human immunoglobulin G (H-IgG) was obtained from Shanghai Sangon Biotech Co., Ltd. (Shanghai, China). Carcinoembryonic antigen (CEA) and Alpha-fetoprotein (AFP) were purchased from Shanghai Linc-Bio Science Co., Ltd. (Shanghai, China). All chemicals were used without further purification.

Whatman chromatography paper #2 (58.0 × 68.0 cm) was received from GE Healthcare Worldwide (Shanghai, China), and it was cut into A4 size before use. Scanning electron microscopic (SEM) analyses were performed using a QUANTA FEG 250 thermal field emission SEM (FEI Co., Portland, USA) equipped with an Oxford X-MAX50 X-ray energy-dispersive spectrometer (EDS) (Oxford Co., Oxford, UK). X-ray photoelectron spectroscopy (XPS) spectra were recorded using an ESCALAB MK II X-ray photoelectron spectrometer. The photocurrent response and electrochemical impedance spectroscopy (EIS) were performed using a CHI 760D electrochemical workstation (Shanghai CH Instruments Co., Shanghai, China) with a strand three-electrode system consisting of the working, reference, and counter electrodes. The EIS experiments were carried out in PBS (pH 7.4, 0.1 M) containing 5.0 mM [Fe(CN)_6_]^3−/4−^ with 0.1 M KCl. Photocurrent response measurements were carried out in PBS (pH 7.4, 0.1 M) at room temperature with 0.1 M AA. During the PEC measurement procedure, a Xe lamp was used as the excitation light source, and the applied potential was 0 V.

### 2.2. Preperation of BiVO_4_ Photoelectrode

The preparation of BiVO_4_ photoelectrode is similar to that stated in previous literature [57]. In brief, the 2.5 mM Bi(NO_3_)_3_·5H_2_O and 3 mM NH_4_VO_3_ were dissolved in 20 mL deionized water and named solution A. Then, the urea solution (13 mM, 10 mL) was added into the solution A under ultrasound for half an hour to acquire a homogeneous solution. After the ultrasound, the prepared solution was transferred into a 100 mL Teflon-lined stainless autoclave with the conductive side of the working tab facing down. The Teflon-lined stainless autoclave was put into the oven at 180 °C for 1 h. Finally, the PWE with a layer of BiVO_4_ was washed with deionized water and dried at 60 °C for 24 h.

### 2.3. Preparation of BiVO_4_/FeOOH Photoelectrode

FeOOH needle-like crystals were modified on the surface of the BiVO_4_ layer by a simple photo-electrodeposition procedure which was reported previously with modification [62]. Briefly, the photo-electrodeposition was carried out in a three-electrode system with 0.1 M Fe_3_O_4_ solution at 0.4 V. The paper-working electrode (PWE) with a layer of BiVO_4_ was used as the working electrode, and the saturated Ag/AgCl and Pt electrodes were applied as reference electrode and counter electrode. After deposition, the as-obtained BiVO_4_/FeOOH photoelectrode was cleaned with DI water and dried at 60 °C overnight.

### 2.4. Design and Analytical Steps of the Dual-Mode Lab-on-Paper Device

**Figure 1:**
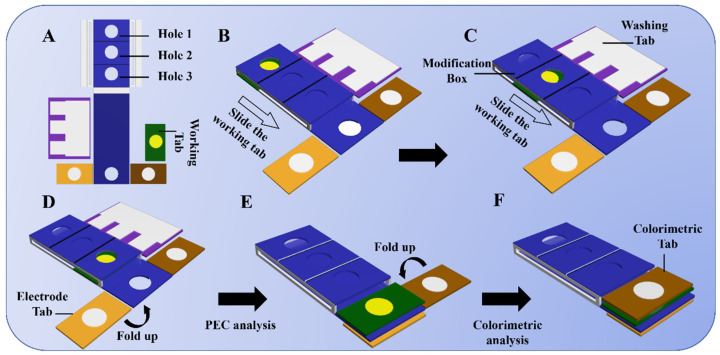
Assembly and operation illustration of the proposed dual-mode lab-on-paper device. (**A**) Unfolded sensor platform, (**B**–**D**) modification process for PWE of the biosensor platform, (**E**) folding process for PEC detection of target, and (**F**) folding process for colorimetric detection of target.

The proposed lab-on-paper was designed with the software of Adobe Illustrator CS4 (Figure S1), and then, printed out with a wax printer on Whatman No. 1 chromatography paper. After obtaining the wax-printed lab-on-paper devices, it was then baked at 200 °C for a few minutes for melting the wax into paper and forming a hydrophobic wall (Figures S2 and S3). The assembly steps of the dual-mode lab-on-paper device from preparation (Figure 1A), modification (Figure 1B–F), to the final detection integrating PEC and colorimetric strategies is elucidated in Figure 1. Since the hydrophobic zone can make the liquid stay in the working area of the circle for a long time, the working area is only at the circle, which is advantageous to achieve a good modification effect for the PWE of the lab-on-paper device. The assembled state of the lab-on-paper dual-mode device is illustrated in Figure 1B. In this state, the working tab designed for modification of the PWE (yellow circle part) is initially put into the modification box to make sure the PWE circle matches well with the hole 1 when the CEA antibodies (Ab) are anchored onto the surface of the prepared BiVO_4_/FeOOH modified onto the PWE of the lab-on-paper device. Subsequently, the working tab is moved from hole 1 to hole 2 to realize the further modification step where the bovine serum albumin (BSA) is introduced onto the flexible lab-on-paper dual-mode device for blocking excess active sites, avoiding nonspecific connection or physical adsorption of molecules (Figure 1C). Then, the working tab is moved towards hole 3 (Figure 1D) which assists in immobilizing the CEA targets onto the interface of the BiVO_4_/FeOOH nanocomposite-functionalized PWE. It is worth noting that the working electrode needs to be placed in the corresponding hydrophilic area of the washing tab after each modification above, so that the waste liquid can be sucked away with the washing tab. After all modification processes are finished, the electrode tab (printed reference electrode and counter electrode) is folded downward to cover the pedestal with the circle on the electrode matching the hole on the side of the pedestal. The working tab is transferred to cover the electrode tab to build a three-electrode conjunction in the meantime. Finally, the proposed sensing platform can be utilized to conduct the PEC analysis in this case (Figure 1E). To achieve the visual colorimetry, the colorimetric tab (a white circle embedded TMB) is folded left to cover the working zone (Figure 1F). After dropped H_2_O_2_, the loaded TMB could be catalyzed to generate blue product, realizing the colorimetric detection of CEA due to the peroxidase-like activity of FeOOH/BiVO_4_. Consequently, the proposed dual-mode lab-on-paper device constructed by combining PEC and colorimetric analysis was efficaciously applied for ingeniously quantifiable dual-mode detection of CEA targets. The matching physical images depicting the analytical steps above are displayed in Figure S4.

### 2.5. PEC/Colorimetric Mechanism of Dual-Mode Lab-on-Paper Biosensing Platform

As is illustrated in Scheme 1, the needle-like FeOOH was deposited on the surface of BiVO_4_ photoactive materials which are in situ grown onto the 3D paper fibers of PWE to enhance the PEC performance of the photoanode due to the unique PEC activities of the prepared BiVO_4_/FeOOH nanocomposites, which is beneficial for increasing the electron transmission rate and expanding the absorption capacity of light, eventually leading to the separation ability of photoinduced carriers. Under light irradiation conditions, the electrons are excited from the valence band to the conduction band of BiVO_4_, and then, the FeOOH layer plays a role of hole transport layer which is beneficial for increasing the hole transmission rate due to the Bi-O-Fe interfacial bond formed and accelerating the separation ability of photoinduced carriers [29,32]. Concretely, along with the deposition of FeOOH, the photogenerated hole in the valence band of BiVO_4_, which would normally recombine with the electron in the surface of the BiVO_4_ layer without the formation of nanocomposites, is preferentially transferred to the FeOOH layer where the hole is further consumed by ascorbic acid (AA). Meanwhile, the photogenerated electron in the conduction band of BiVO_4_ is transferred to the paper-based electrode, eventually producing the improved performance of PEC signal readout of the prepared lab-on-paper device, as shown in the Scheme S1 in the Supplementary Materials part. Moreover, by simply folding the colorimetric tab embedded with TMB, the colorimetric detection signal output can be achieved in the presence of H_2_O_2_, due to the enzyme-like catalytic activity of FeOOH, which catalyzes the TMB to generate blue oxidized TMB in the presence of H_2_O_2_. Thus, after the PEC detection is finished, the corresponding colorimetric result could be obtained to realize the dual-mode operation of the lab-on-paper device. As the concentration of CEA increases, the PEC signal will reduce because the more CEA bounded to the surface of BiVO_4_/FeOOH through the specific reaction, the less compounds will be irradiated, and the colorimetric signal will also decrease because there will be more CEA anchored on the surface of electrode hindering catalytic oxidation of TMB. Accordingly, the ultrasensitive detection of CEA targets is realized by the proposed lab-on-paper device with the PEC and colorimetric signals readout.

## 3. Results

### 3.1. Morphology and Structure

**Figure 2:**
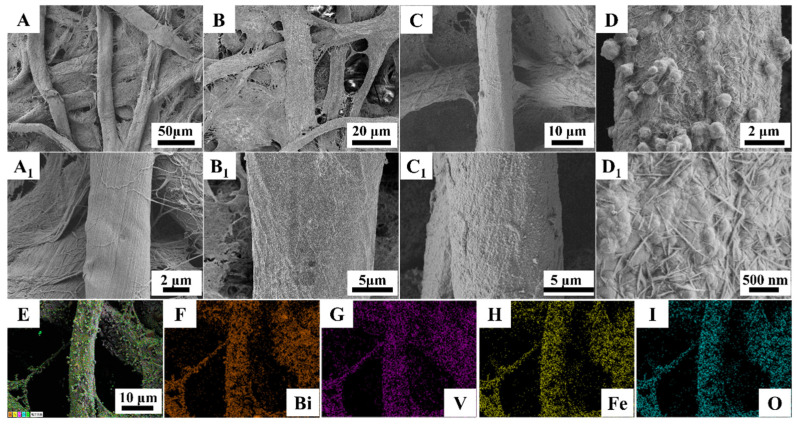
(**A**,**A_1_**) SEM images of bare cellulose fiber with different magnifications. (**B**,**B_1_**) SEM images of cellulose fiber modified with Au NPs. (**C**,**C_1_**) SEM images of paper-based BiVO_4_ under different magnifications. (**D**,**D_1_**) SEM images of paper-based BiVO_4_/FeOOH under different magnifications. (**E**–**I**) The EDS elemental mapping images for Bi, V, Fe, and O elements.

To demonstrate the successful synthesis of the BiVO_4_/FeOOH photoelectrode, the scanning electron microscopy (SEM) was characterized. Figure 2 presents the morphologies of the as-prepared BiVO_4_/FeOOH nanocomposites onto the surface of cellulose paper at various modification stages. The unmodified bare paper electrode consisting of crisscross 3D paper fibers inside the paper substrate with a relatively smooth surface was observed (Figure 2A,A_1_), providing the properties of modification of high surface area functional nanomaterials onto the PWE. Figure 2A_1_ presents a single cellulose paper fiber of the intricate cellulose fibers. Then, the uniform Au NPs were introduced to the bare insulated paper electrode to endow the contact surface with superior conductivity (Figure 2B,B_1_). After the BiVO_4_ modification, the PWE was densely coated with an occasionally protruding grain BiVO_4_ layer (Figure 2C,C_1_). When the FeOOH needle-like crystals were further grown on its surface by a simple photo-electrodeposition synthesis, the obvious FeOOH nanoneedle arrays functionalized BiVO_4_ were observed (Figure 2D,D_1_), forming the BiVO_4_/FeOOH nanocomposites on each single cellulose fiber of PWE, which enables it to achieve a good performance of the proposed lab-on-paper device by facilitating the reactants’ access to the biosensing interface, eventually improving the sensitivity of the biosensing platform. The length of the FeOOH needle-like crystals was about 300–700 nm, and the diameter was approximately 35 nm (Figure 2D_1_). To further confirm the successfully synthesized paper-based BiVO_4_/FeOOH photoelectrode, energy-dispersive spectrum (EDS) elemental mapping was utilized to characterize its elemental contents. As shown in Figure 2E–I, the uniform distribution of Bi, V, Fe, and O elements can be clearly observed in the EDS elemental mapping images, indicating the successful preparation of BiVO_4_/FeOOH nanocomposites onto the PWE as the photoelectrode for the proposed lab-on-paper device. Based on these SEM images and the EDS element mapping analysis, it is demonstrated that the BiVO_4_/FeOOH photoelectrode was successfully fabricated.

**Figure 3:**
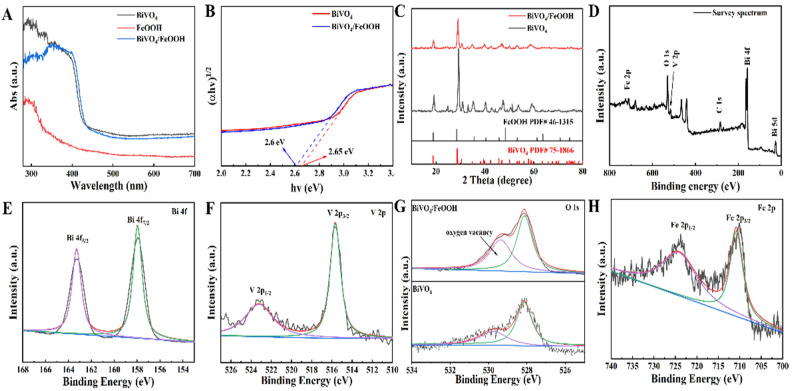
(**A**) UV-vis absorption spectrum of BiVO_4_, FeOOH, and BiVO_4_/FeOOH. (**B**) The corresponding Tauc plots of (**A**). (**C**) XRD patterns of BiVO_4_, FeOOH, and BiVO_4_/FeOOH. (**D**–**H**) XPS spectrum of the BiVO_4_/FeOOH nanocomposite.

Besides the optical property of the BiVO_4_ layer, the FeOOH nanoneedles and BiVO_4_/FeOOH nanomaterials were studied by solid UV-vis diffuse reflectance absorption spectra (Figure 3A). FeOOH showed almost no absorption of visible light due to its broad band gap, and the absorption edge was about 380 nm. Additionally, FeOOH has an absorption peak from about 290 nm. Pristine BiVO_4_ and BiVO_4_/FeOOH exhibit analogous absorbance edges at 440 nm; BiVO_4_/FeOOH also has an absorption peak from about 290 nm, and compared to the Pristine FeOOH, the BiVO_4_/FeOOH nanomaterials have a significantly enhanced absorption of visible light. This suggests that BiVO_4_ still plays the important role in the light harvest efficiency. The band gap energies (Eg) of the BiVO_4_ and BiVO_4_/FeOOH were estimated by the Tauc plots, which were shown to be 2.65 and 2.6 eV (Figure 3B), respectively. Those results suggested that the formation of BiVO_4_/FeOOH composites contributed efficient carrier separation and strengthened the absorbing capacity of visible light, which made it probable to benefit and boost the light-to-current transition ability [63]. Figure 3C presents the XRD analysis of BiVO_4_ and BiVO_4_/FeOOH. In contrast with the JCPDS card of BiVO_4_ (PDF#75-1866) and FeOOH (PDF#46-1315) [64,65], it could be demonstrated that the BiVO_4_/FeOOH was synthesized successfully. Interestingly, no significant added peaks can be seen between the BiVO_4_ and BiVO_4_/FeOOH, suggesting that the FeOOH layer deposited on the surface of BiVO_4_ was too thin to be detected by XRD. Moreover, the XPS technique was also performed to verify the fabricated BiVO_4_/FeOOH. Figure 3D shows the survey spectrum of BiVO_4_/FeOOH, certifying the existence of Bi, V, O, and Fe in BiVO_4_/FeOOH. As depicted in Figure 3E, the diffraction peaks that appeared at 163.2 eV and 157.8 eV are ascribed to the Bi_4f5/2_ and Bi_4f7/2_ orbitals of Bi^3+^ in BiVO_4_/FeOOH composites. The V_2p_ orbitals are fitted at 523.4 and 515.9 eV, ascribing to the V_2p1/2_ and V_2p3/2_ orbitals of V^5+^ in BiVO_4_, as exhibited in Figure 3F. [66] The O_1s_ spectrum is deconvoluted into two peaks around 529.7 eV and 528.1 eV (Figure 3G). Based on them, the peaks at 529.7 eV and 528.1 eV are ascribed to the oxygen vacancy (O_V_) species and lattice oxygen (O_L_), respectively. The relative ratio of O_V_/O_L_ was calculated based on peak areas in Table S1, and the results are 0.41 for BiVO_4_ and 0.77 for BiVO_4_/FeOOH. As depicted in Figure 3H, the Fe_2p_ peaks are incorporated from the two peaks of Fe_2p1/2_ (724.3 eV) and Fe_2p3/2_ (710.7 eV), and the existence of Fe_2p_ peaks in the BiVO_4_/FeOOH composite demonstrate the successful composition of FeOOH onto the surface of BiVO_4_ [67].

### 3.2. PEC and Electrochemical Properties of the Proposed Sensing Platform during Modification

**Figure 4:**
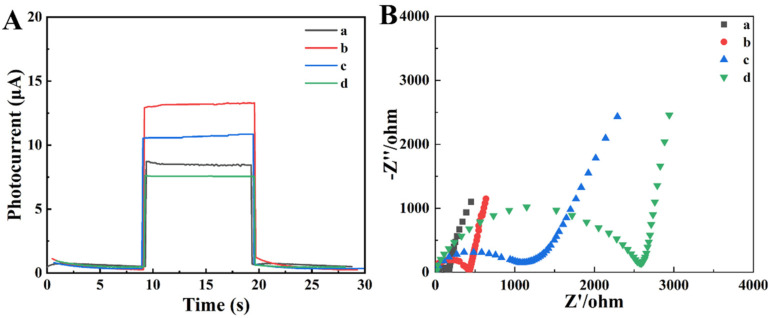
(**A**) Photocurrent responses of the modification of PWE in PBS (0.1 M, pH 7.4) containing 0.1 M AA: (a) BiVO_4_, (b) BiVO_4_/FeOOH, (c) BiVO_4_/FeOOH/Ab, (d) BiVO_4_/FeOOH/Ab/BSA. (**B**) Electrochemical impedance spectroscopy (EIS) for modification process of the proposed sensing platform in PBS (0.1 M, pH 7.4) containing 5.0 mM [Fe (CN)_6_]^3−/4−^ and 0.1 M KCl: (a) BiVO_4_, (b) BiVO_4_/FeOOH, (c) BiVO_4_/FeOOH/Ab, (d) BiVO_4_/FeOOH/Ab/BSA.

As depicted in Figure 4A, the preparation of BiVO_4_/FeOOH/Ab/BSA stage by stage was primarily measured based on the photocurrent performances with chronoamperometry under alternate ray illumination. Compared with only BiVO_4_ (curve a), an obviously more powerful photocurrent of BiVO_4_/FeOOH (curve b) was exhibited under irradiation, adequately certifying the enhancement of the separation efficiency of the photogenerated carrier transmission resistance and facilitating the charge transfer by the formation of BiVO_4_/FeOOH composite. After finishing embellishment with Ab and BSA onto the BiVO_4_/FeOOH, a downward direction of the PEC signal intensity of the prepared photoelectric chemical electrode emerges with BiVO_4_/FeOOH/Ab (curve c) and BiVO_4_/FeOOH/Ab/BSA (curve d), on account of the electric insulation of proteins which could restrain sacrificial agent propagation to the BiVO_4_/FeOOH surface and ulteriorly descend the PEC signal intensity. What is more, the surface state of the proposed PEC biosensor during modification was also following tested ground on electrochemical impedance spectroscopy (EIS). In Figure 4B, the size of the semicircle visually showed the transfer resistance of electrons (*R_et_)*. The bare BiVO_4_ exhibited the smallest semicircle (curve a, *R_et_* = 198.67 Ω) attributed to the higher photoelectric property. With the introduction of FeOOH (curve b, *R_et_* = 487.4 Ω), the *R_et_* increased because of the mean conductivity of semiconductors. After finishing incubation of Ab onto the surface of BiVO_4_/FeOOH composites, the *R_et_* value enlarged more (curve c) due to the inferior electron transmission capacity. Furthermore, after BSA was introduced to the BiVO_4_/FeOOH/Ab surface to shield the redundant active sites, the value of *R_et_* sustained an increase (curve d), resulting from the inferior conductivity of the small organic molecule. Consequently, the measured EIS performances of each modification procedure pointed out the successful building of the paper-based PEC device.

### 3.3. PEC and Colorimetric Dual-Mode Readout Analytical Performance of CEA

**Figure 5:**
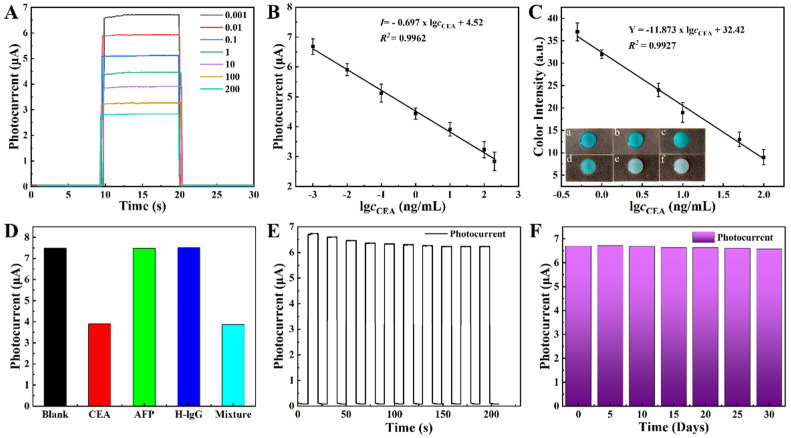
(**A**) Photocurrent signals of different concentrations of CEA from 0.001 to 200 ng·mL^−1^. (**B**) The calibration curve of (**A**). (**C**) Calibration curve and practical images (inset) of colorimetric detection mode with different concentrations from 0.1, 1, 5, 10, 50, to 100 ng·mL^−1^. (**D**) Selectivity of the proposed biosensing platform for the interferences (10 ng·mL^−1^): the blank, CEA, AFP, H-lgG and Mixture. (**E**) Stability of the proposed lab-on-paper device at 0.001 ng·mL^−1^ for 200 s. (**F**) The photocurrent response of PEC biosensor incubated with 0.001 ng·mL^−1^.

To evaluate the performance of the proposed lab-on-paper device, the experimental optimization conditions of the constructed biosensing platform were first investigated, including pH of electrolyte, and incubation time of CEA, which is shown in the Supplementary Materials (Figure S5). Under the optimized construction conditions, the sensitivity of the proposed lab-on-paper for CEA sensing was employed by performing photocurrent signals and color intensity after incubating CEA of diverse concentrations. As presented in Figure 5A, the photocurrent signals within 10 s-20 s illustrate a reduced trend with CEA concentrations increasing from 0.001 to 200 ng·mL^−1^ under the optimal condition, which is wider than other reported PEC biosensors (Tables S2 and S3). The linear equation was *I* = −0.697 × lg*c*_CEA_ + 4.52 (*R^2^* = 0.9962) and the limit of detection (LOD) was obtained to be 0.0008 ng·mL^−1^ (Figure 5B). The color intensity exhibited dependence on CEA content in Figure 5C. The linear equation was *Y* = −11.873 × lg*c*_CEA_ + 32.42 (*R^2^* = 0.9927), and the LOD was 0.013 ng·mL^−1^. Meanwhile, the illustration a-f in Figure 5C depicts the processes when the color shifted from blue to pale blue as the CEA concentration increased (0.5–100 ng·mL^−1^). To evaluate the specificity performance of the proposed PEC platform on CEA sensing, the photocurrent of the fabricated biosensor incubated with different interferents was measured under the same conditions as indicated in Figure 5D. A visibly decreased photocurrent signal with the presence of CEA was obtained and other interfering markers of cancer made no difference on the PEC performance; however, the mixture of CEA, AFP, and H-lgG indicated obvious reduction the same as the presence of CEA, which showed us the proposed sensing platform has excellent specificity for CEA. To evaluate the stability manifestation of the prepared sensor, the photocurrent was recorded under light turned on and off for 200 s (Figure 5D). After 10 light on-and-off cycles, the photocurrent response could still reach 92% of the initial photocurrent, presenting the fine stability of the constructed platform. Additionally, the stability of the proposed lab-on-paper was also investigated after long-term storage. To explore the effectiveness of long-term storage on the constructed biosensor platform, the prepared biosensor was stored at 4 ℃ and their photocurrent ws measured with incubated 0.001 ng·mL^−1^ CEA every 5 days (Figure 5F). After storing for 30 days, the photocurrent values measured by the constructed biosensing platform could remain at 97% compared to the initial signal value, which further makes clear to us the credible storage stability of the constructed paper-based biosensor.

### 3.4. The Application of Proposed Lab-on-Paper for Real Serum Samples

The application of the prepared PEC biosensing platform was verified by detecting real serum samples. In a word, the diluted human serum samples (collected by centrifugation from whole blood) were added to different concentrations of CEA for the investigation of the real assay performance of the prepared PEC biosensor. As shown in Table 1, the recovery rates of prepared biosensor ranged from 96.7% to 103% and the RSD was 1.1–3.2% indicating the good accuracy and performance of the PEC analysis platform.

## 4. Conclusions

In this work, the dual-mode lab-on-paper device was successfully constructed for sensitive detection of CEA and realized by the BiVO_4_/FeOOH nanocomposites in situ grown onto cellulose fibers for a PEC and colorimetric signals readout system. The fabricated photoelectrode with BiVO_4_/FeOOH nanomaterials was characterized by SEM, XRD, UV-vis, and XPS techniques. On account of the good PEC performance and desirable enzyme-like catalytic activity of BiVO_4_/FeOOH, the creditable PEC and colorimetric signals were successfully recorded for sensitive detecting of the CEA targets. What is more, according to the artful design of the lab-on-paper sensing platform, the as-prepared lab-on-paper device has integrated PEC and colorimetric detection technology on paper substrate, achieving a simple but efficient portable device for dual-mode detection of CEA. We believe that the proposed strategy holds good potential applications in biosensing, especially the point-of-care diagnosis.

## Figures and Tables

**Table 1:** Recovery experiments of the proposed PEC biosensor.

Serum Sample	Added, ng·mL^−1^	Found, ng·mL^−1^	Recovery, %	RSD, %
1	1 × 10^−2^	1.01 × 10^−2^	101	1.1
2	1 × 10^−1^	9.7 × 10^−2^	97	2.7
3	1 × 10^0^	1.03 × 10^0^	103	1.8
4	1 × 10^1^	9.67 × 10^0^	96.7	3.2
5	1 × 10^2^	9.83 × 10^1^	98.3	2.3

## Data Availability

Not applicable.

## Supplementary Materials

The following supporting information can be downloaded at: https://www.mdpi.com/article/10.3390/bios13010103/s1. Preparation of Au modified paper working electrode (PWE); modification process of the working electrode; the optimization of experimental conditions; Scheme S1: Schematic diagrams of the proposed mechanism of photoinduced carrier behaviors in BiVO_4_/FeOOH photoelectrode; Figure S1: Wax pattern (A) and corresponding size (B) schematic layout of lab-on-paper device based on 3D printing technology; Figure S2: Wax-patterns of lab-on-paper device on a paper sheet (A4) before baking; Figure S3: Wax-patterns of lab-on-paper device on a paper sheet (A4) after baking; Figure S4: The physical picture of modification and detection of proposed biosensor; Figure S5: Effect of (A) The pH of buffer on photocurrent responses of biosensor (*c*_CEA_ = 0.1 ng·mL^−1^); (B) The incubation time of CEA (*c*_CEA_ = 0.1 ng·mL^−1^); Table S1: The relative ratio of O_V_/O_L_ in BiVO_4_, and FeOOH/BiVO_4_ photoelectrodes Table S2: Comparison of other PEC-based CEA biosensors [68,69,70,71]; Table S3: Comparison of other methods-based CEA biosensors [1,72,73,74].
