# Supplementary material for: PEC/Colorimetric Dual-Mode Lab-on-Paper Device via BiVO4/FeOOH Nanocomposite In Situ Modification on Paper Fibers for Sensitive CEA Detection
Source: Biosensors (Basel). 2023 Jan 6;13(1):103. doi: 10.3390/bios13010103 (PMC9855910; doi:10.3390/bios13010103)
Supplement: Supplementary file 1 [file biosensors-13-00103-s001.zip › biosensors-2113407-supplementary.pdf]

biosensors

MDPI

Supplementary Material

PEC/Colorimetric Dual-mode Lab-on-paper Device via $BiVO_4$/FeOOH Nanocomposites In Situ Modification on Paper Fibers for Sensitive CEA Detection

Xu Li [1,†], Jiali Huang [1,†], Jiayu Ding [1], Mingzhen Xiu [2], Kang Huang [2], Kang Cui [1,2,*], Jing Zhang [1], Shiji Hao [3], Yan Zhang [1,4,*], Jinghua Yu [1] and Yizhong Huang [2,*]

[1] School of Chemistry and Chemical Engineering, University of Jinan, Jinan 250022， China
[2] School of Materials Science and Engineering, Nanyang Technological University, Singapore 639798, Singapore
[3] School of Materials Science & Engineering, Dongguan University of Technology, Guangdong 523808, China
[4] Key Laboratory of Optic-electric Sensing and Analytical Chemistry for Life Science, MOE, Qingdao University of Science and Technology, Qingdao 266042, China
* Correspondence: chm_cuik@ujn.edu.cn (K. C.); chm_zhangyan@ hotmail.com (Y. Z.); yzhuang@ntu.edu.sg (Y. H.); .
† These authors contributed equally to this work.

Content

Preparation of Au modified paper working electrode (PWE)..2

Modification process of the working electrode 2

The optimization of experimental conditions 2

Scheme S1. Schematic diagrams of the proposed mechanism of photoinduced carrier behaviors in $BiVO_4$/FeOOH photoelectrode 3

Figure S1. Wax pattern (A) and corresponding size (B) schematic layout of lab-on-paper device based on 3D printing technology. 3

Figure S2. Wax-patterns of lab-on-paper device on a paper sheet (A4) before baking 4

Figure S3. Wax-patterns of lab-on-paper device on a paper sheet (A4) after baking ...5

Figure S4. The physical picture of modification and detection of proposed biosensor 6

Figure S5. Effect of (A) The pH of buffer on photocurrent responses of biosensor (c_{CEA} = 0.1 ng·mL^{-1}); (B) The incubation time of CEA (c_{CEA} = 0.1 ng·mL^{-1}). 6

Table S1 The relative ratio of O_V/O_L in $BiVO_4$, and FeOOH/$BiVO_4$ photoelectrodes. 7

Table S2 Comparison of other PEC-based CEA biosensors 7

Table S3 Comparison of other methods-based CEA biosensors 7

Preparation of Au modified paper working electrode (PWE)

The Au/paper substrate of lab-on-paper device was obtained by growing Au NPs layer on the cellulose fibers. Herein, 40 mL of ultrapure water was put into a round-bottom flask and heated it to 90 °C, and 0.4 mL of 1% $HAuCl_4$ solution was dripped to the prepared solution and then heated to 96 °C for 1 min. Next, 1.4 mL of 1% trisodium citrate was added and stirred for 20 min. Finally, the above solution was stirred and cooled to receive a gold seed solution. After that, 40 μL of hydroxylamine hydrochloride and gold seed solution (volume ratio 1:1) was dropped on the surface of paper substrate each time and dried at air. After repeated dripping five times, it was thoroughly washed with ultrapure water and dried at 60 °C. The Au modified paper electrode was prepared successfully.

Modification process of the working electrode

Briefly, first, a layer of Au nanoparticles (NPs) was grown on the surface of cellulose fiber to enhance the conductivity of fiber. Second, the $BiVO_4$/FeOOH heterojunction was modified to the surface of Au NPs. Next, CS solution (15 μL, 0.1 wt%) was dropped to electrode surface and dried at room temperature, and washed by NaOH and DI water, respectively. Then, the GLD (30 μL, 5%) solution was dropped onto the electrode which occur cross-linking reaction with CS for half an hour, following by washed with DI water to remove nonspecific binding GLD. Then, Ab (25 μL, 100 $\mu g \cdot mL^{-1}$) was conjugated to the electrode and place in refrigerator at 4°C for 24 h to full reaction. After that, the electrode was washed with PBS and dropped BSA (25 μL, 1%) at 37 °C for 0.5 h to block excess active sites. The electrode was rinsed with PBS and restored at 4 °C for the subsequent measurement of PEC signals. Finally, to prepare the colorimetric detection, the TMB (20 mM, 20 μL), acetic acid (pH 4.5, 20 μL) and 10 μL PBS (pH 7.4, 0.1 M) were embedded in the color area.

The optimization of experimental conditions

To attain the best analytical performance of the PEC immunosensor, the PEC measurement conditions were carefully optimized, including the pH of electrolyte, and incubation time of CEA. All the optimization experiments were carried out by PEC signal in PBS containing 0.1 M AA. From the Figure S5, to obtain the optimal pH value of the PBS, we investigated the PEC signal in PBS (0.1 M) with different pH. As is shown in Figure S5A, the peak photocurrent ascended with the increase of the pH of PBS, followed by the photocurrent drop off with the pH value increased. And the maximum photocurrent is about 5.1 μA at pH 7.4. The optimal incubation time of CEA indicated in Figure S5B, the photocurrent decreased with the increasing incubation time of CEA and remained a steady value after 40 min. A longer incubation time did not enhance the PEC signal of the proposed biosensor. Therefore, to save detection time, 40 min was utilized for the reaction between Ab and target CEA (c_{CEA} = 0.1 $ng \cdot mL^{-1}$). The above results show that the optimum pH, and incubation time are 7.4, and 40 min, respectively.

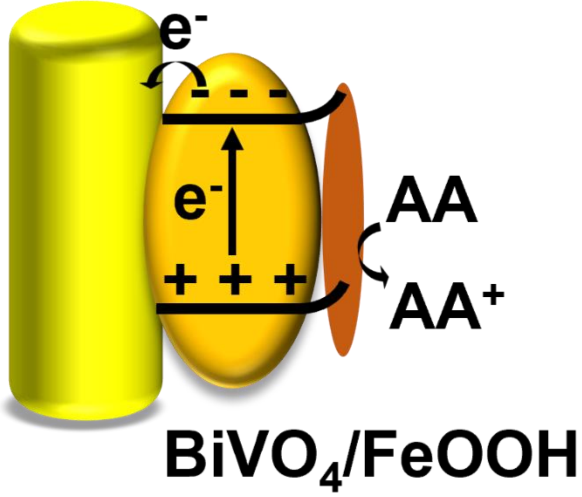

Scheme S1. Schematic diagrams of the proposed mechanism of photoinduced carrier behaviors in $BiVO_4$/FeOOH photoelectrode.

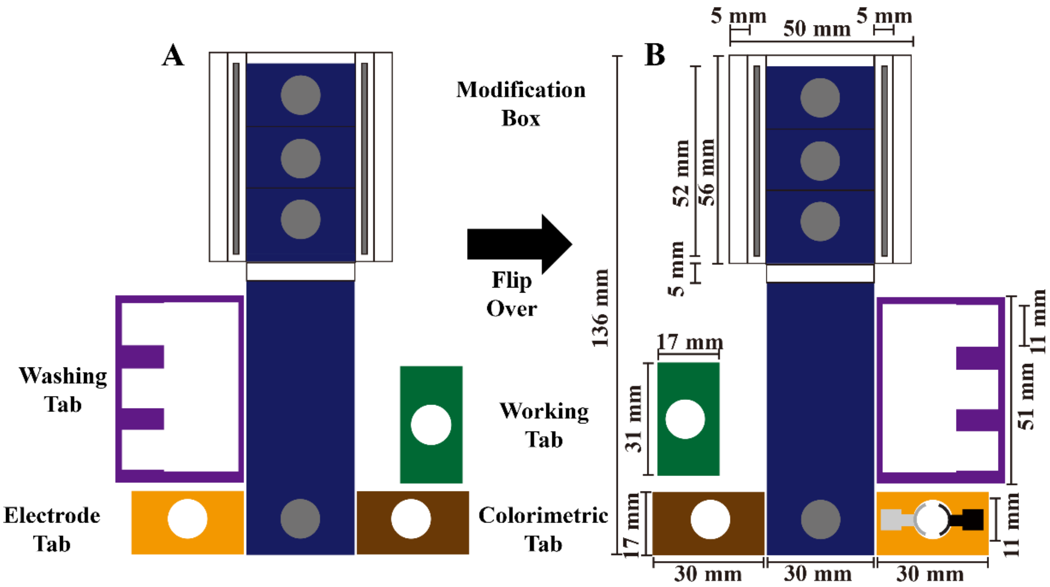

Figure S1. Wax pattern (A) and corresponding size (B) schematic layout of lab-on-paper device based on 3D printing technology.

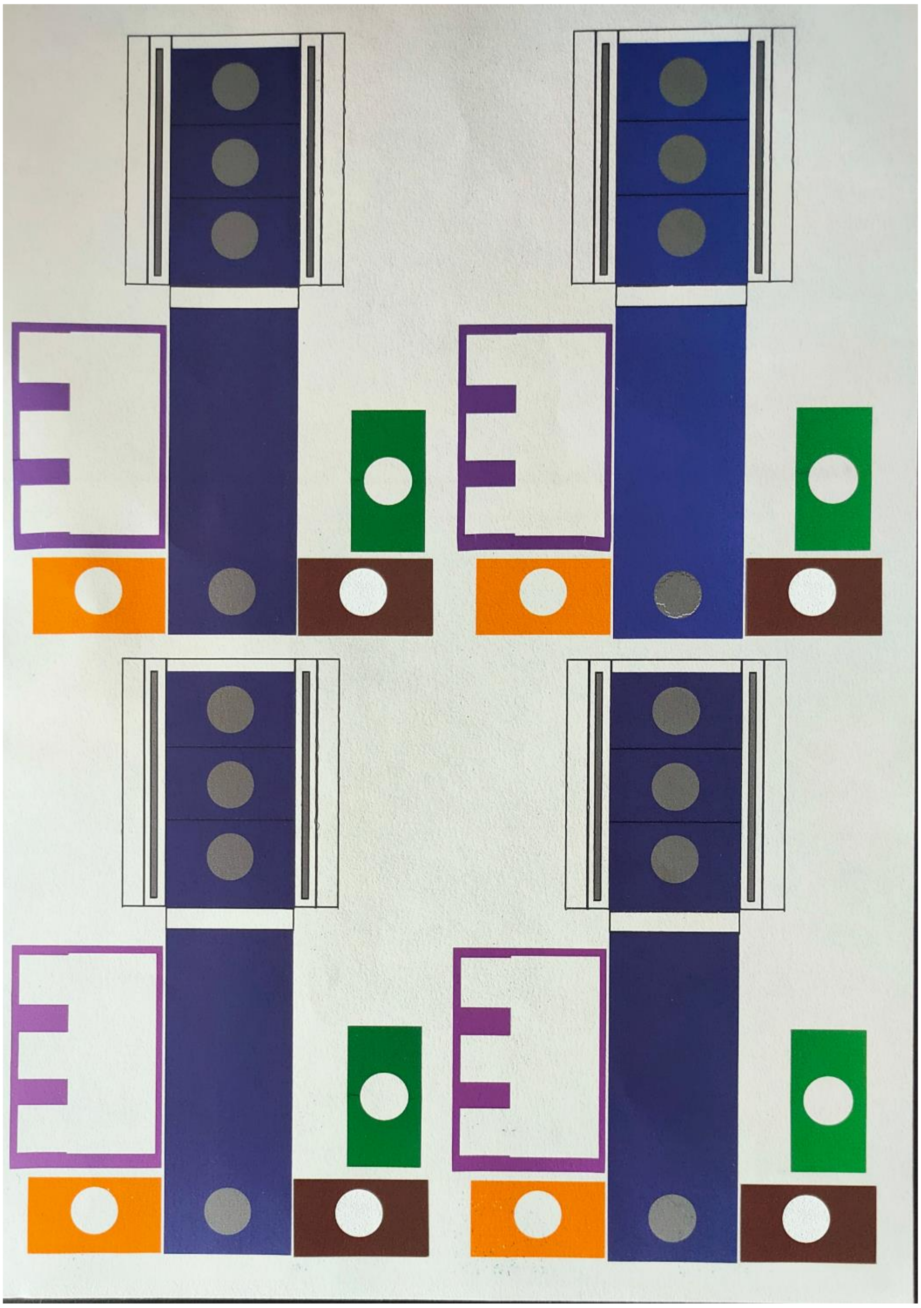

Figure S2. Wax-patterns of lab-on-paper device on a paper sheet (A4) before baking.

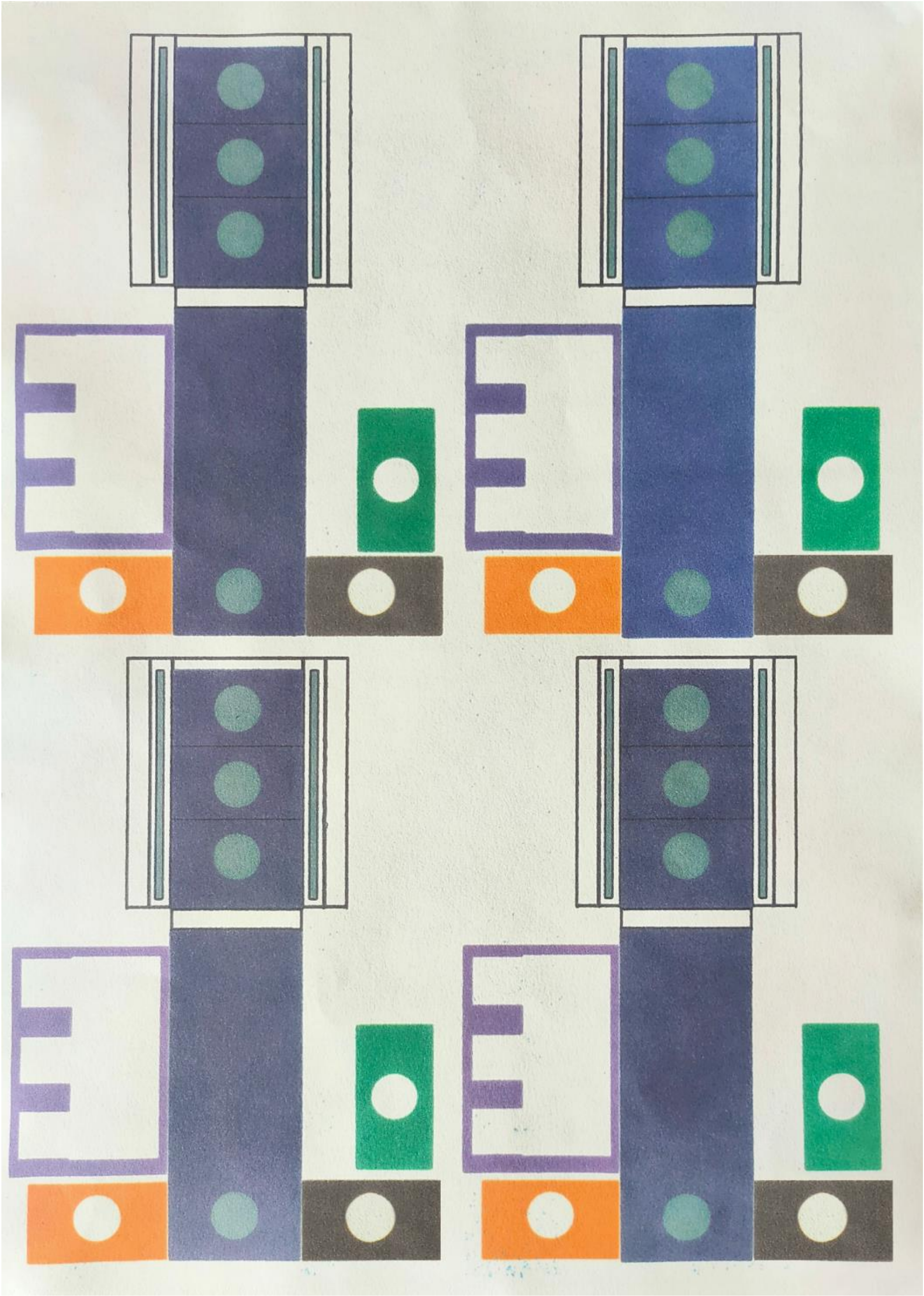

Figure S3. Wax-patterns of lab-on-paper device on a paper sheet (A4) after baking.

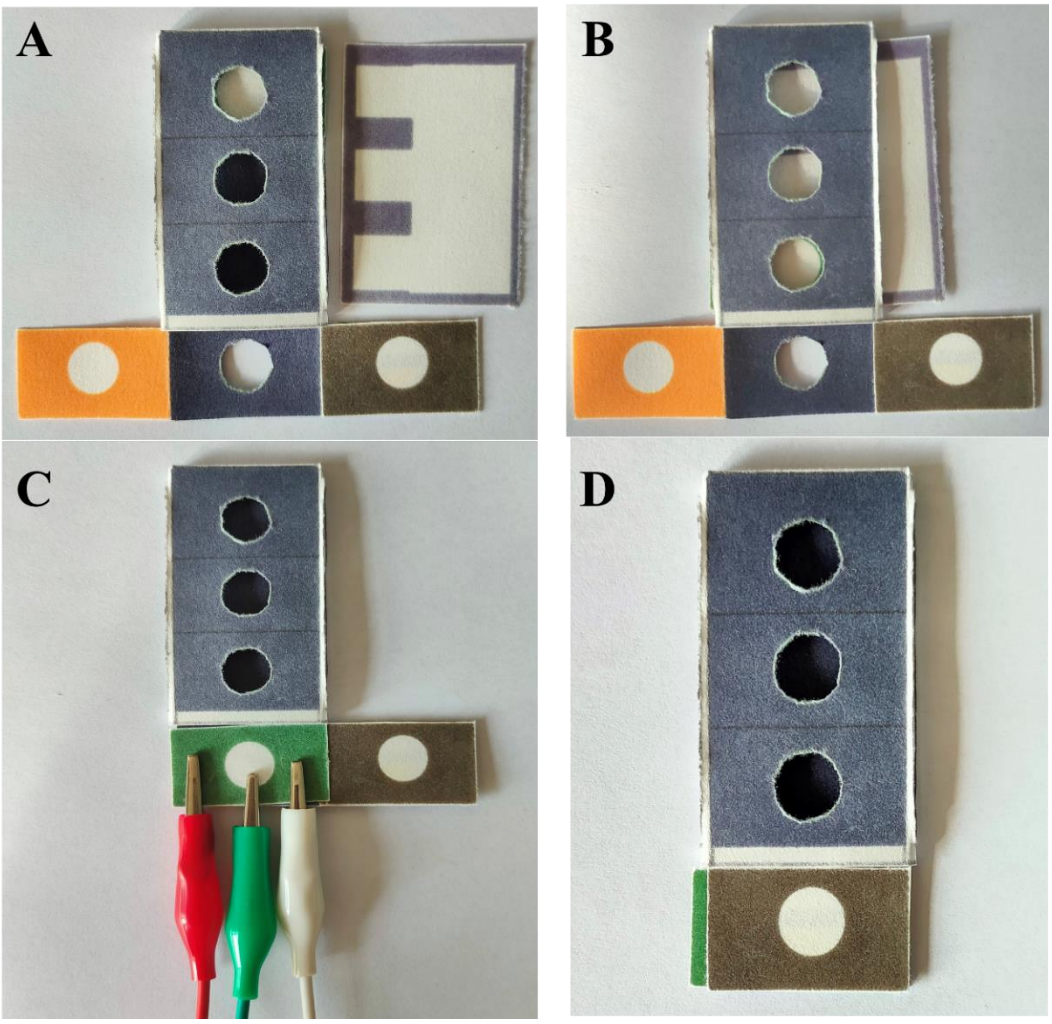

Figure S4. The physical picture of modification and detection of proposed biosensor.

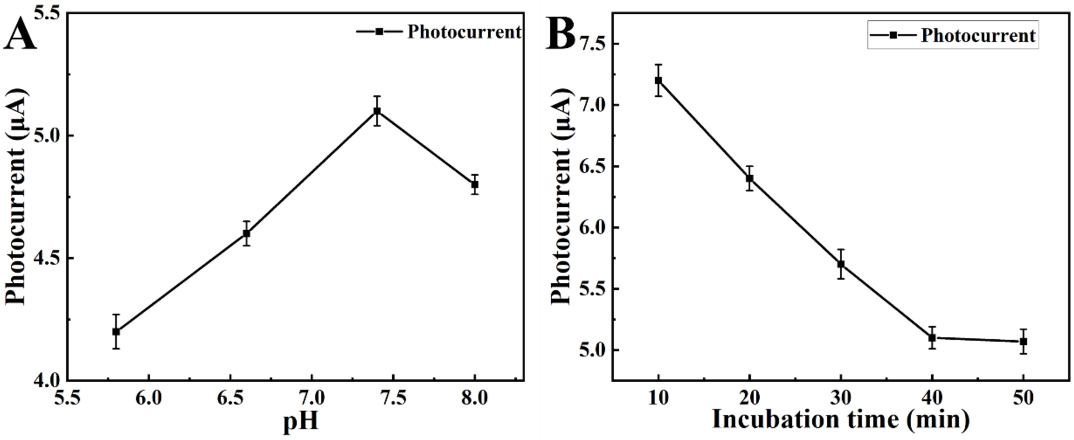

Figure S5. Effect of (A) The pH of buffer on photocurrent responses of biosensor (c_{CEA} = 0.1 ng·mL^{-1}); (B) The incubation time of CEA (c_{CEA} = 0.1 ng·mL^{-1}).

Table S1. The relative ratio of O_V/O_L in $BiVO_4$, and FeOOH/$BiVO_4$ photoelectrodes.

Photoelectrode	Peak area of lattice oxygen (O_L)	Peak area of oxygen vacancy (O_V)	Relative ratio of O_V/O_L
$BiVO_4$	7623.44	3125.61	0.41
FeOOH/$BiVO_4$	9738.44	7520.08	0.77

Table S2. Comparison of other PEC-based CEA biosensors.

Material	Detection range (ng·mL^{-1})	LOD (ng·mL^{-1})	Ref.
ZIF-8-assisted $NaYF_4$:Yb,Tm@ ZnO	0.1-300	0.032	1
HCdS@Au nanosphere	0.015-2.4	0.0035	2
In_2O_3/$CdIn_2S_4$	0.01-50	0.0028	3
C_3N_4/$CuInS_2$	0.02-40	0.0052	4
$BiVO_4$/FeOOH	0.001-200	0.0008	this work

Table S3. Comparison of other methods-based CEA biosensors.

Method	Detection range (ng·mL^{-1})	LOD (ng·mL^{-1})	Ref.
chemiluminescence	0.1-64	0.085	5
electrochemistry	0.05-20	0.01	6
fluorescence	1-40	0.3	7
electrochemiluminescence	0.005-500	0.00167	8
photoelectrochemical	0.001-200	0.0008	this work

Reference

1. Lv, S., Zhang, K., Zhu, L. and Tang, D., ZIF-8-Assisted $NaYF_4$:Yb,Tm@ZnO Converter with Exonuclease III-Powered DNA Walker for near-Infrared Light Responsive Biosensor. *Anal. Chem.* **2020,** *92*, 1470-1476.
2. Zhou, Y., Lv, S., Wang, X. Y., Kong, L. and Bi, S., Biometric Photoelectrochemical-Visual Multimodal Biosensor Based on 3D Hollow HCdS@Au Nanospheres Coupled with Target-Induced Ion Exchange Reaction for Antigen Detection. *Anal. Chem.* **2022,** *94*, 14492-14501.
3. Huang, X., Lin, Q., Lu, L., Li, M. and Tang, D., $In_2O_3/CdIn_2S_4$ Heterojunction-Based Photoelectrochemical Immunoassay of Carcinoembryonic Antigen with Enzymatic Biocatalytic Precipitation for Signal Amplification. *Anal. Chim. Acta* **2022,** *1228*, 340358.
4. Zhang, K., Lv, S., Zhou, Q. and Tang, D., CoOOH Nanosheets-Coated G-$C_3N_4/CuInS_2$ Nanohybrids for Photoelectrochemical Biosensor of Carcinoembryonic Antigen Coupling Hybridization Chain Reaction with Etching Reaction. *Sens. Actuators B Chem.* **2020,** *307*, 127631.
5. Mao, Y., Wang, N., Yu, F., Yu, S., Liu, L., Tian, Y., Wang, J., Wang, Y., He, L. and Wu, Y., Simultaneous Detection of Carcinoembryonic Antigen and Neuron-Specific Enolase in Human Serum Based on Time-Resolved Chemiluminescence Immunoassay. *Analyst* **2019,** *144*, 4813-4819.
6. Gu, X., She, Z., Ma, T., Tian, S. and Kraatz, H. B., Electrochemical Detection of Carcinoembryonic Antigen. *Biosens. Bioelectron.* **2018,** *102*, 610-616.
7. Chen, Y., Guo, X., Liu, W. and Zhang, L., Paper-Based Fluorometric Immunodevice with Quantum-Dot Labeled Antibodies for Simultaneous Detection of Carcinoembryonic Antigen and Prostate Specific Antigen. *Mikrochim. Acta* **2019,** *186*, 112.
8. Li, N. L., Jia, L. P., Ma, R. N., Jia, W. L., Lu, Y. Y., Shi, S. S. and Wang, H. S., A Novel Sandwiched Electrochemiluminescence Immunosensor for the Detection of Carcinoembryonic Antigen Based on Carbon Quantum Dots and Signal Amplification. *Biosens. Bioelectron.* **2017,** *89*, 453-460.
